# 

**Published:** 1852-10

**Authors:** 


### CONTENTS

### OF NO. IV.

### ORIGINAL COMMUNICATIONS.

### ESSAYS AND CASES.

Art. I.—Observations on Excision of the Superior Maxillary Bone; illustrated by five Cases. By S. D. Gross, M. D., Professor of Surgery in the Medical Department of the University of Louisville. - - - - - - 277

Art. II.—Case of Doubtful Paternity. By W. L Sutton, M. D., President of the Medical Society of Kentucky. 308

### REVIEWS.

Art. III.—The Principles and Practice of Surgery. Illustrated by three hundred and sixteen engravings on wood. By William Pirrie, F. R. S. E., Regius Professor of Surgery in the Medical College and University of Aberdeen; Surgeon to the Royal Infirmary, etc., etc. Edited with additions by John Neill, M. D., Surgeon to the Pennsylvania Hospital, Demonstrator of Anatomy in the University of Pennsylvania, etc., - - - - 314

Art. IV.—Transactions of the Medical Association of Southern Central New York, at the Annual Meeting held at Owego, June, 1852. - - - - - - - - 331

Art. V.—Transactions of the Missouri Medical Association of the State of Missouri, at its Second Annual Meeting, June, 1852. - - - - - - - - - - - - 336

SELECTIONS FROM FOREIGN AND HOME JOURNALS.

Cancerous Degeneration of Warts, - - - - - - 337
Relapsing Fever, - - - - - - - - - - 353
Liberality of a Foreign Critic, - - - - - - - 355
The Reciprocal Influence of Acute Diseases and Menstruation, 356
Muscular Power of the Insane, - - - - - - - - 357
On the General Improvement in the condition of the Insane, - 358
Obstinate Intermittent Coryza—Instantaneous Cure, - - 358
American Institute of Homœopathy, - - - - - - 359
Improved Syringe, - - - - - - - - - - 360

ORIGINAL INTELLIGENCE.

University of Louisville, - - - - - - - - 361
Kentucky Medical Society, - - - - - - - 361
Dr. Samuel A. Cartwright, - - - - - - - 362
New Medical Schools, - - - - - - - - - 364
Professional Appointments, - - - - - - - 364
Poisonous Chloroform, - - - - - - - - - 365
Transylvania Medical Journal, - - - - - - - 367
Cholera and Dysentery, - - - - - - - - - 367
Obituary, - - - - - - - - - - - - 368
Medical Feuds, - - - - - - - - - - - 368

